# A Retrospective Analysis of the COVID-19 Pandemic Evolution in Italy

**DOI:** 10.3390/biology10040311

**Published:** 2021-04-08

**Authors:** Anna Fochesato, Giulia Simoni, Federico Reali, Giulia Giordano, Enrico Domenici, Luca Marchetti

**Affiliations:** 1 Fondazione The Microsoft Research—University of Trento, Centre for Computational and Systems Biology (COSBI), 38068 Rovereto, Italy; fochesato@cosbi.eu (A.F.); simoni@cosbi.eu (G.S.); reali@cosbi.eu (F.R.); domenici@cosbi.eu (E.D.); 2 Department of Mathematics, University of Trento, 38123 Trento, Italy; 3 Department of Industrial Engineering, University of Trento, 38122 Trento, Italy; giulia.giordano@unitn.it; 4 Department of Cellular, Computational and Integrative Biology, University of Trento, 38123 Trento, Italy

**Keywords:** COVID-19, retrospective analysis, disease prevention, health policy, computational models, SIDARTHE model

## Abstract

**Simple Summary:**

Given the progress of the COVID-19 pandemic, it has become crucial to retrace the past epidemic trajectories to grasp non-trivial, qualitative features of viral dynamics that could contribute to the design of general guidelines for future outbreaks or epidemics. In this regard, we used a refinement of the SIDARTHE (Susceptible, Infected, Diagnosed, Ailing, Recognized, Threatened, Healed, Extinct) model to develop a retrospective computational analysis focused on an Italian case study. Our work aimed at evaluating the efficacy of adopted countermeasures (inferred from the resulting model parameters), and additionally providing an estimate of the undetected viral circulation as well as the day zero of the COVID-19 outbreak in Italy, which are not directly inferable from the data.

**Abstract:**

Late 2019 saw the outbreak of COVID-19, a respiratory disease caused by the new coronavirus SARS-CoV-2, which rapidly turned into a pandemic, killing more than 2.77 million people and infecting more than 126 million as of late March 2021. Daily collected data on infection cases and hospitalizations informed decision makers on the ongoing pandemic emergency, enabling the design of diversified countermeasures, from behavioral policies to full lockdowns, to curb the virus spread. In this context, mechanistic models could represent valuable tools to optimize the timing and stringency of interventions, and to reveal non-trivial properties of the pandemic dynamics that could improve the design of suitable guidelines for future epidemics. We performed a retrospective analysis of the Italian epidemic evolution up to mid-December 2020 to gain insight into the main characteristics of the original strain of SARS-CoV-2, prior to the emergence of new mutations and the vaccination campaign. We defined a time-varying optimization procedure to calibrate a refined version of the SIDARTHE (Susceptible, Infected, Diagnosed, Ailing, Recognized, Threatened, Healed, Extinct) model and hence accurately reconstruct the epidemic trajectory. We then derived additional features of the COVID-19 pandemic in Italy not directly retrievable from reported data, such as the estimate of the day zero of infection in late November 2019 and the estimate of the spread of undetected infection. The present analysis contributes to a better understanding of the past pandemic waves, confirming the importance of epidemiological modeling to support an informed policy design against epidemics to come.

## 1. Introduction

COVID-19 was identified in China in late 2019 and spread worldwide so rapidly that the World Health Organization declared the pandemic alert on 11 March 2020 [1]. Italy was among the first Western countries hit by the virus, with an official recognized case dated back to 21 February 2020. From that day, a national monitoring system for COVID-19 cases was established to track the virus spread and monitor the epidemic evolution. Informed by daily data on new infection cases and hospitalizations, the Italian government set countermeasures that were sufficiently timely and stringent to slow down the epidemic curve and ease the national healthcare system. In particular, the two-month lockdown in spring 2020, albeit harmful for the economy [2], was crucial in curbing the initial exponential spread and in reducing the transmissibility rate. The softer policies that followed (gradual re-opening of all activities and free circulation), paving the way for a pre-epidemic number of social contacts, boosted the infection cases again from early October on, driving the reproduction rate of SARS-CoV-2 back to alarming values.

In such a public health emergency, mathematical models represent a valuable approach to gain a rigorous data-driven understanding of the disease, to analyze dynamical properties, and to evaluate future scenarios. Within this framework, a retrospective computational analysis of the epidemic evolution can increase our knowledge of specific COVID-19 features through a quantitative/qualitative interpretation of the events. Many researchers have proposed models for COVID-19, building upon the common SIR (Susceptible, Infectious, Recovered)-SEIR (Susceptible, Exposed, Infectious, Recovered) models for human-to-human transmission, to study the key traits of the epidemic [3,4,5,6]. In this work, we opted for the SIDARTHE (Susceptible, Infected, Diagnosed, Ailing, Recognized, Threatened, Healed, Extinct) model [7,8], which captures the different epidemiological stages of the infection due to its granularity in stratifying infected subjects for both symptoms and detection. We used a refined version of the model to capture the COVID-19 trajectory fuelled by the original SARS-CoV-2 strain in Italy and to assess the efficacy of adopted countermeasures, and the smooth operation of healthcare and contact tracing systems in terms of model rates. In addition, we dated the actual onset of the Italian epidemic and we gained non-trivial insights into the dynamics of undetected infection, which is crucial to establish an efficient contact tracing system. For our work, we limited the studied period to 24 February until 20 December 2020 to leave aside confounding factors due to the start of the Italian vaccination campaign on 27 December 2020 and to the emergence of mutated SARS-CoV-2 strains, documented from mid-December [9]. Indeed, the qualitative evaluation of the efficacy of the adopted countermeasures in terms of the model rates would have been distorted by the higher fitness capacity and virulence associated with the new variants [10,11,12].

**Figure 1:**
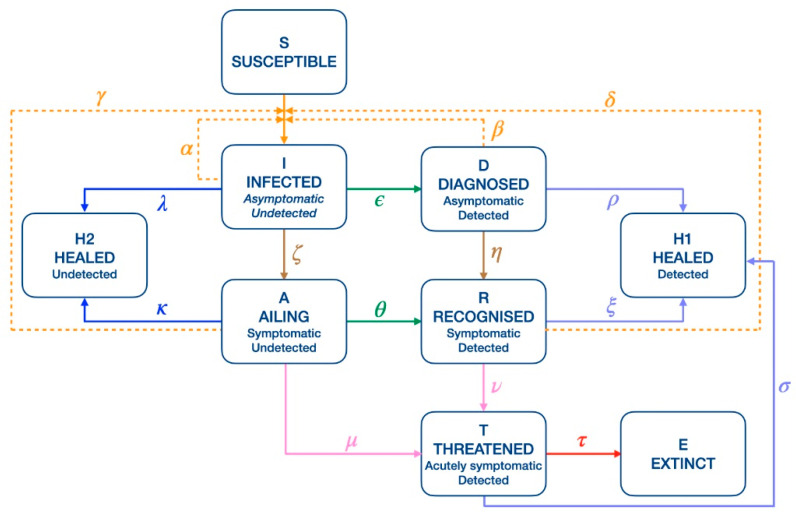
Network of the model. We considered the SIDARTHE (Susceptible, Infected, Diagnosed, Ailing, Recognized, Threatened, Healed, Extinct) model [7] and divided the original healed class H into H1 and H2, representing the healed individuals from the detected and undetected classes, respectively. Continuous lines represent the flow of individuals from one class to another. Dashed lines represent the contagion of susceptible individuals due to one of the infected class. The color code of the lines is associated with the biological meaning of the parameters: orange for contagion, green for diagnosis, dark and light blue for healing, brown for the development of symptoms, pink for the development of critical conditions and red for death. Model parameters are: α (contagion rate due to undetected asymptomatic), β (contagion rate due to detected asymptomatic), δ (contagion rate due to detected symptomatic), γ (contagion rate due to undetected symptomatic), ε (diagnosis rate for undetected asymptomatic), θ (diagnosis rate for undetected symptomatic), λ (healing rate for undetected asymptomatic), ρ (healing rate for detected asymptomatic), κ (healing rate for undetected symptomatic), ξ (healing rate for detected symptomatic), σ (healing rate for life-threatened cases), ζ (rate of symptom development for undetected asymptomatic), η (rate of symptom development for detected asymptomatic), μ (rate of critical condition development for undetected symptomatic), ν (rate of critical condition development for detected symptomatic), and τ (death rate).

## 2. Materials and Methods

### 2.1. Mathematical Model

For our study, we used a refined version of the SIDARTHE model [7,8], whose graphical representation is provided in Figure 1. The original model divides the entire population into eight mutually exclusive compartments describing different infection stages: each individual can be either susceptible (S), asymptomatic undetected or pauci-symptomatic infected (I), detected asymptomatic infected (D), undetected symptomatic infected (A), detected symptomatic infected (R), detected life-threatened symptomatic infected (T), recovered (H), or dead (E). Our refinement further partitioned the recovered patients into those who had been previously detected (H1) and those who had not (H2), so as to ease the parameter calibration on the available data. Therefore, we considered the closed-form system of differential equations:

$$\begin{array}{l} \frac{dS}{dt}=-S\left(t\right)\left[\alpha I\left(t\right)+\;\beta D\left(t\right)+\;\gamma A\left(t\right)+\;\delta R\left(t\right)\right];\\ \frac{dI}{dt}=S\left(t\right)\left[\alpha I\left(t\right)+\;\beta D\left(t\right)+\;\gamma A\left(t\right)+\;\delta R\left(t\right)\right]-\left(\varepsilon +\zeta +\lambda \right)I\left(t\right);\\ \frac{dD}{dt}=\varepsilon I\left(t\right)-\left(\eta +\;\rho \right)D\left(t\right);\\ \frac{dA}{dt}=\zeta I\left(t\right)-\left(\theta +\;\mu +\kappa \right)A\left(t\right);\\ \frac{dR}{dt}=\eta D\left(t\right)+\theta A\left(t\right)-\left(\nu +\xi \right)R\left(t\right);\\ \frac{dT}{dt}=\mu A\left(t\right)+\nu R\left(t\right)-\left(\sigma +\tau \right)T\left(t\right);\\ \frac{dH1}{dt}=\rho D\left(t\right)+\xi R\left(t\right)+\sigma T\left(t\right);\\ \frac{dH2}{dt}=\lambda I\left(t\right)+\kappa A\left(t\right);\\ \frac{dE}{dt}=\tau T\left(t\right). \end{array}$$

In agreement with the results of the sensitivity analysis on the loss of immunity presented in the original SIDARTHE model [7], our model neglected the possibility of re-infections within the considered time horizon. In fact, in [7], the authors showed that the introduction of relapses into the SIDARTHE model did not significantly affect the trajectories, apart from the number of recovered individuals.

### 2.2. Model Parameter Calibration

To estimate the model parameters, we used the covariance matrix adaptation evolution strategy (CMA-ES) [13] relying on daily data (detected cases, hospitalized patients, ICU (Intensive Care Unit) patients, recovered, and deaths) from 24 February to 20 December 2020. The CMA-ES is a derivative-free method for non-linear/non-convex optimization problems, which belongs to the class of evolutionary algorithms. Inspired by biological principles, these algorithms iteratively generate candidate solutions by stochastically modifying previous estimates to achieve better fitness, i.e., a better objective function value.

We defined a tailored fitting strategy, which retraces the shifting policies enforced in the analyzed time interval by updating the model parameter estimates. Indeed, we argue that the latter are not constant over time, but rather evolve in response to changes in policies (lockdown, partial closures, and re-openings) and behaviors (e.g., frequent hand- washing and wearing protective face masks). To mark the updating intervals accordingly with the interventions, we accounted for the Italian National Decrees enforced on 1, 11, 22 March, 14 April, 4 and 18 May, 25 October, and 29 November, as well as for the changes in the swab policy dated 28 March. Given the frequent, minor amendments to policies that occurred in summer and part of fall 2020, we selected a reduced number of updating days (two days in the 18 May–25 October interval and one day in the 25 October–29 November interval) to be optimized within the global procedure to model those periods parsimoniously. Therefore, CMA-ES estimated both the model parameters and the three parametric days within a single optimization procedure, which aimed at fitting the model to the complete time series. As a result, we calibrated the model parameters for the first time interval (24 February–1 March) and for every subsequent one identified as follows: 1–11 March, 11–22 March, 22–28 March, 28 March–14 April, 14 April–4 May, 4–18 May, 18 May–first optimized day, first optimized day–second optimized day, second optimized day–25 October, 25 October–third optimized day, third optimized day–29 November, and 29 November–20 December. After the first interval, the model parameters were not estimated from scratch, but rather resulted from the estimation of multiplicative scaling factors in the range 0.5–1.5 applied to the parameter values of the previous interval. In this way, the estimates of the model parameters in subsequent intervals are analytically related, reflecting a continuous adaptation of their values over time.

We informed the optimization algorithm with prior knowledge on standard epidemic mechanisms and on the specific features of COVID-19 to constrain the parameter search space. First, we assumed that undetected asymptomatic cases are more likely to spread the epidemic than both undetected symptomatic cases, which are supposed to stay at home to recover, and detected asymptomatic/symptomatic cases, which are assumed to be quarantined (*α* > *γ* > *δ* and *β*, see Figure 1). Second, we assumed the same infection rate for the two classes of detected cases (*δ* = *β*), since they both undergo forced social isolation, and they are therefore equally (un)likely to spread the virus. Third, we imposed the probability of detection to be higher for infected patients with visible symptoms (*θ* > *ε*), and, finally, we supposed the same healing rate for the asymptomatic classes (*λ* = *ρ*) and for the symptomatic ones (*κ* = *ξ*) since we argued that disease severity, and hence recovery, are not sensitive to detection. Moreover, we set the initial conditions for the undetected cases to be greater than those for the detected cases and, following the findings reported in [14], we imposed R0 to not exceed the value of 6. Finally, we fixed the rates of symptom development for both undetected and detected asymptomatic (ζ and η parameters) to 0.125 to model an incubation period of 8 days, in agreement with [15].

**Figure 2:**
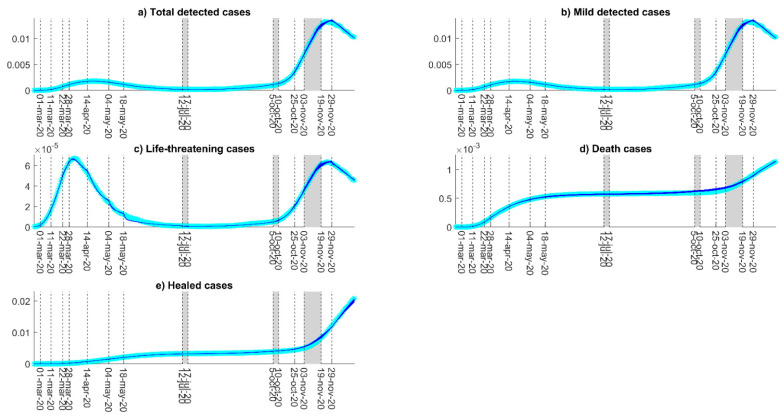
Model calibration results. (**a**) Daily evolution of total (asymptomatic, symptomatic, and life-threatening) detected infection cases, (**b**) daily evolution of mild (asymptomatic and symptomatic) detected cases, (**c**) daily evolution of life-threatening cases, (**d**) cumulative evolution of deaths, (**e**) cumulative evolution of healed-detected cases. All panels represent the 3-day moving average of the data with light-blue asterisks and the model simulations obtained from the repeated calibrations with blue lines. Both data and model dynamics are normalized as fractions of the Italian population. Dashed vertical lines represent policies that marked a fitting update, while gray-shaded rectangles span the area for optimized updates in the 25 repeated model calibrations.

**Figure 3:**
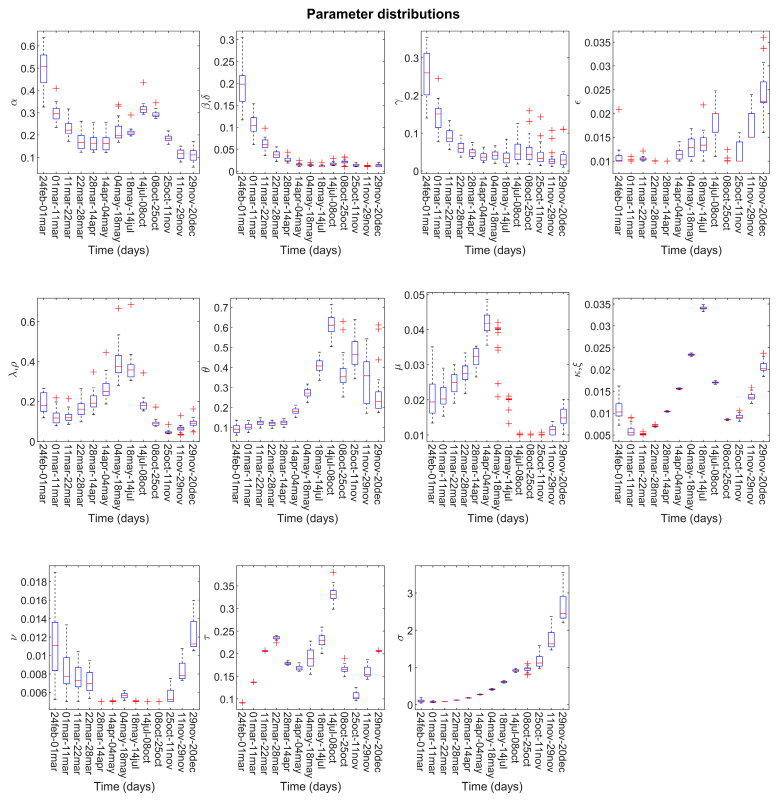
Model parameter distribution. For each estimated parameter, we reported the estimate distribution across the multiple calibrations at each update. Model parameters are *α* (contagion rate due to undetected asymptomatic), *β* (contagion rate due to detected asymptomatic), *δ* (contagion rate due to detected symptomatic), *γ* (contagion rate due to undetected symptomatic), *ε* (diagnosis rate for undetected asymptomatic), *θ* (diagnosis rate for undetected symptomatic), *λ* (healing rate for undetected asymptomatic), *ρ* (healing rate for detected asymptomatic), *κ* (healing rate for undetected symptomatic), *ξ* (healing rate for detected symptomatic), sigma (healing rate for life-threatened cases), *ζ* (rate of symptom development for undetected asymptomatic), *η* (rate of symptom development for detected asymptomatic), *μ* (rate of critical condition development for undetected symptomatic), *ν* (rate of critical condition development for detected symptomatic), and *τ* (death rate).

We imposed a range of 10^−2^–1 for the model parameter estimates (except for *κ*, *ν*, and *α*, for which we fixed a lower bound of 5 × 10^−3^), and a range of 10^−9^–10^−4^ for the initial model variables. We assumed a uniform prior distribution for the model parameters within the assumed ranges, and we set the maximum number of iterations equal to 100 times the squared number of the parameters to be calibrated. We computed the model calibration 25 times to provide a robust distribution of the model parameter estimates. Each calibration provided different estimations of the model parameters due to the random start of the CMA-ES algorithm. We highlighted the posterior distribution of the three parametric days with gray strips in Figure 2 and that of the model rates with the boxplots of Figure 3.

## 3. Results

Our protocol for the parameter estimation of the refined SIDARTHE model accurately captured the two-wave trend displayed by the COVID-19 epidemics in the analyzed time period, as Figure 2 shows. The low variance in the selection of the optimized updating days in summer and fall (gray strips in Figure 2, see Materials and Methods) confirmed the bias reduction of our strategy and its robustness to multiple calibrations. Interestingly, our results suggest that the updating day selection was deeply informed during the optimization by relevant interventions acting on the data shape. Indeed, we found that the first day of update consistently occurred in mid-July, when international flights were shortly earlier re-admitted with mild impositions on quarantine for arrivals, while the second one occurred in early October, following school reopening. This finding could suggest how these two events could have affected the trajectory pattern, though with a delay of around two weeks from their start, and might have contributed to the second COVID-19 outbreak. On the contrary, the optimized update in November displayed the highest variance, since no leading events occurred in that period to drive the day selection. This finding may indicate that countermeasures implemented in November smoothly affected the reduction of COVID-19 driving factors.

The distribution of the model parameter estimates (see Figure 3) provides further insight into the efficacy of adopted countermeasures and into the epidemic management by the healthcare and the testing systems. Further insight into the efficacy of the adopted countermeasures can be deduced by analyzing the computed distribution of model parameter estimates displayed in Figure 3, which can be also informative of how the healthcare system and detection campaign managed the virus spread (see Figure 3). The double U-shape-like trend estimated for the infectious rate of asymptomatic undetected (α parameter) highlighted the beneficial effects of the spring lockdown and of fall target policies in curbing the virus spread. Indeed, the above interventions constantly decreased the infection rate estimates, with a sharper drop as long as the policy persisted. In late spring/summer, as the lockdown ended and its effects started to vanish, the model captured an evident increase of the infectious parameter, which could have been a signal for a second wave to come. Interestingly, the comparison across the estimates for such a parameter from 6 July to 25 October 2020 seemed to suggest that wearing face masks with no exceptions when outdoor, a behavioral measure compulsorily reintroduced in early October, played an active role in reducing the transmission of the virus. Moreover, the model suggests how the experience in treating patients gained during the epidemic had an overall positive impact on recovery rates. However, the second outbreak put pressure on hospitals, negatively affecting the recovery rates and positively affecting the rate of critical condition development for symptomatic detected patients (ν parameter). In a similar way, the model calibration suggests that the detection parameters were sensitive to the swab demand. Indeed, even though the diagnosis rates exhibited an increasing behavior on average, reflecting a smoother operation of the testing system during the epidemic, the estimates for the initial part of both waves were lower (ε and θ parameters). This could be explained by a rapid saturation of the testing system due to the insufficient resources and to the tremendous number of daily COVID-19 cases (>40,000), respectively.

**Figure 4:**
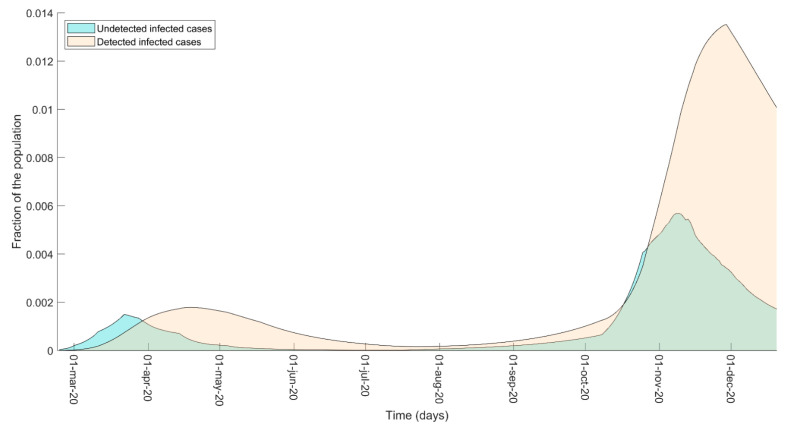
Undetected–detected comparison. Comparison of the undetected infected cases (model variables I and A) and of the detected infected cases (variables D, R, and T) estimated by the model up to 20 December 2020. The predicted boost in the number of undetected cases is associated with the epidemic expansion phase, when the system for testing and contact tracing fails due to the exponential increase of new infected.

Since the spread of the COVID epidemic is fueled by the infections of the undetected cases [16,17], which by definition cannot be retrieved from the data, we used the model to shed light on the interplay between detected and undetected cases (Figure 4). The model seemed to relate the decline of the undetected-detected ratio, up to its reversal in late March, to the effects of hard social isolation and of the established swab campaign during the first wave. In addition, the model seemed to confirm that the undetected cases increase when the epidemic expansion saturates the system for testing and contact tracing, which is fundamental to control the epidemic spread. Moreover, for the analyzed part of the second wave, the model captures an increasing capacity in detecting and tracing infected cases, which may be a consequence of a better organization of the testing system due to the expected new outbreak. In this regard, the predominance of detected cases over the undetected, with the resulting large-scale isolation of subjects more likely to spread the virus, could explain why soft policies alone were still quite effective in controlling the second wave before the emergence of new SARS-CoV-2 variants of concern.

**Figure 5:**
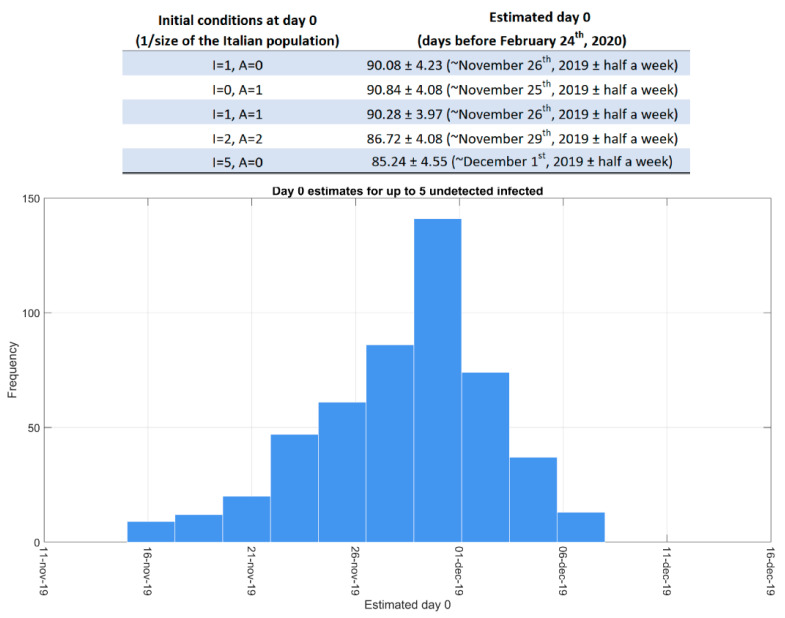
Estimates of the epidemic day 0 in Italy. All the combinations of asymptomatic (I) and symptomatic (A) undetected cases involving at most 5 infected cases were tested to predict the day 0 of the epidemic outbreak in Italy. We used each parameter set resulting from repeated calibrations to make the prediction. The histogram shows the estimate distribution for all the described tests, while the table collects the mean and the standard deviation of the day 0 estimate for a representative subset of initial conditions. In the simulations, all the model variables, except I and A, were initialized to 0.

Finally, we zoomed in our analysis on the early stage of the epidemic. In agreement with [18], our model predicted an undetected–detected ratio of 10:1 on 24 February 2020, suggesting that COVID-19 might have been circulating in Italy, though unknown, far before the identification of the first case [19,20]. This hypothesis seems to be supported, among the others, by the presence of IgM/IgG antibodies against SARS-CoV-2 nucleocapsid protein in blood samples collected in Milan at the real beginning of the outbreak [21]. In this regard, we argue that such an unrecognized circulation of the virus, mainly driven by asymptomatic undetected cases, could have fuelled the exponential spread, which occurred in late February/early March. Hence, we performed “shooting”-forward simulations to estimate the day 0 of the Italian epidemic, assuming that at that day only a few infected cases (at most 5) were present. The results obtained set the first COVID-19 evidence in Italy back to late November–early December 2019 (see Figure 5), in agreement with [22,23].

## 4. Conclusions

We used a refined version of the SIDARTHE model to perform an analysis that retraces the main traits of the Italian epidemic when only the original strain of SARS-CoV-2 was present. Our work revealed hidden properties of the dynamics and qualitatively interpreted the countermeasures in terms of data shape and parameters. In this regard, we could confirm the beneficial effect of behavioral and lockdown policies (non-pharmaceutical interventions) as the first line of defence against COVID-19, in accordance with [24]. Our computational approach provided insight into the number of undetected cases over time: their number, which could not be directly taken from data, could be estimated due to the structure of the model, which accounts for the distinction between diagnosed and non-diagnosed infection cases. The comparison between the numbers of undetected and detected cases can be used to assess the performance of the testing and contact tracing system over time and to inform the stringency of adopted policies. In this regard, the second outbreak could be representative, with a predominance of detected cases over undetected ones, allowing softer policies to slow down the wave. Moreover, the model dynamics of undetected cases supported the intuition of an initial unrecognized spread of COVID-19 that could have fuelled the first outbreak. Such an unidentified virus circulation has hampered the dating of the first COVID-19 cases in Italy, constantly modified in accordance with new findings. In this regard, we approached the issue from a computational point of view, estimating the actual day 0 to be dated back to late November/early December 2019, in accordance with other clinical evidences [22,23]. Among the limitations of our investigation, our results represent the overall Italian situation, without considering that each region had a different epidemic curve, different properties, and response to countermeasures. Even considering these limitations, our analysis provides valuable retrospective insights into the pandemic evolution and into its key properties, as well as complementary information, not explicitly measurable from data. All the provided insights contribute to increasing our knowledge on COVID-19 and supporting an informed policy design to contain COVID-19 and future epidemics in any country.

## Data Availability

Publicly available datasets were analyzed in this study. These data can be found here: https://github.com/pcm-dpc/COVID-19 (accessed on 4 March 2021).
